# Real-Life Experience of the Prognostic Significance of the Primary Tumor Location on the Timing of Colorectal Liver Metastases: A Retrospective Analysis

**DOI:** 10.7759/cureus.30607

**Published:** 2022-10-23

**Authors:** Carlo Signorelli, Pietro Maria Amodio, Mario Giovanni Chilelli, Roberto Santoro, Marta Schirripa, Teresa Valentina Ranalli, Gloria Pessina, Julio Rodrigo Giron Berrios, Federica Natoni, Antonella Virtuoso, Francesca Primi, Marco Mazzotta, Fabrizio Nelli, Agnese Fabbri, Eleonora Marrucci, Enzo Maria Ruggeri

**Affiliations:** 1 Department of Oncology and Hematology, Hospital of Belcolle, Azienda Sanitaria Locale Viterbo, Viterbo, ITA; 2 Department of Surgery, Hospital of Belcolle, Azienda Sanitaria Locale Viterbo, Viterbo, ITA; 3 Department of Molecular Biology, Hospital of Belcolle, Azienda Sanitaria Locale Viterbo, Viterbo, ITA

**Keywords:** hepatic metastases, metachronous liver metastases, synchronous liver metastases, colorectal cancer, primary tumor location

## Abstract

Background

Numerous research studies have looked into how the primary tumor location (PTL) affects patients’ prognosis for colorectal cancer (CRC). Our research aimed to investigate the prognostic effects of PTL in patients with synchronous (SM) and metachronous (MM) colorectal cancer liver metastases (CRCLM).

Material and methods

From 2016 to 2021, we looked back at the records of patients at our institute who were affected by CRCLM.

Results

109 patients were included, of whom 21.1% received CRCLM resection (R0=73.9%), with 57.7% having left-sided colon cancer (LCC) and 42.2% having right-sided colon cancer (RCC). SM predominated (69.7%). The median duration of follow-up was 21,3 months (95%CI=15,4-25,2). ≥5 hepatic metastases prevailed in the SM group (N=61; 83.5%). 21% of all patients underwent CRCLM resection (R0=78.2%). We observed a double rate of patients unresponsive to standard systemic antineoplastic treatments in the SM group (35.8% vs. 17.9% of the MM group) (p=0.27). We found a significantly longer median overall survival (OS) in patients with MM-LCC compared with the other groups (27.7 months; HR=0.3797; 95%CI=0.19-0.74; p=0.0205). The median OS, regardless of PTL, was higher in the MM group (16,5 months vs. 16,1 months; HR=0,29; 95%CI=0,13-0,67; p=0.0038) as well as progression-free survival (PFS) (11 months vs. 10,2 months; HR=0,61; 95%CI=0,33-1,12; p=0.11). Finally, in patients undergoing liver surgery, a noteworthy median OS was shown to be significantly in favor of patients with metachronous liver metastases from the primary left tumor (37.0 months; HR=0.47; 95%CI=0.11-1.96; p=0.0041).

Conclusions

Our real-life study demonstrated that patients with LCC, particularly MM-LCC, have the highest survival and that the timing of CRCLM should be a prognostic factor.

## Introduction

Colorectal cancer (CRC) is one of the major causes of cancer-related morbidity and mortality, ranking third in terms of incidence (1931590 new cases, 10% of all cancer cases) and second in terms of cancer death (935173 deaths, 9.4% of all cancer mortality) globally in 2020 [1]. As the selection and sequencing of treatments vary depending on the patient's presentation, the number of sites where metastases have spread, their location, and their potential for surgical resection, the multidisciplinary approach to the management of metastatic CRC is essential in improving overall survival (OS) for patients with this condition [2]. Current down-staging response rates for this disease are over 50% thanks to a variety of treatment approaches, including systemic chemotherapy and immunotherapy [3]. For patients with colorectal cancer liver metastases (CRCLM), a combination of surgical and non-surgical multimodal therapy offers the best chance of recovery.

The discovery of liver metastases at the time of primary tumor diagnosis has significant therapeutic implications, both in terms of surgical approach and the planning of oncologic systemic treatment, given recent improvements in CRC and CRCLM treatment [4]. The most successful treatment with the possibility for long-term survival and cure is liver resection, notwithstanding recent improvements in the overall management of patients with CRCLM]. The most effective treatments based on the tumor's genetic subtype may soon be determined using molecular signatures [5]. After hepatectomy for CRCLM, five-year OS rates ranged from 47-60%, according to data from numerous trials [6]. However, after hepatic resection, recurrence occurs in 40-75% of patients [7], and 50% of these cases involve the liver [8]. After liver recurrence, repeated hepatectomy has been demonstrated to be feasible and to increase survival [9]. In terms of the normal progression of CRC, the liver is crucial both anatomically and physiologically. At the time of diagnosis, roughly 20% of patients have synchronous metastases, frequently in the liver, and about 35% of patients experience metastases even after receiving curative treatment.

The OS of patients with metastatic CRC has increased significantly over the past ten years, reaching an average of about 30 months thanks to the availability of more and better surgical techniques, more and more potent medicines, and a variety of local ablative treatments. The primary investigation and reporting of synchronous vs. metachronous detection or incidence of CRCLM as a prognostic factor occurred in surgical case series [10]. However, there is no agreement on how synchronous and metachronous are to be defined in the context of CRCLM. For patients with CRC, having synchronous and metachronous CRCLM appears to be a significant prognostic indicator [11]. Before or during surgery on the primary tumor, or within six months after surgery, synchronous liver metastases (SM) are found. On the other hand, metachronous liver metastases (MM) are detected at least six months after the excision of the primary tumor and have a better prognosis [12].

The effect of PTL on the prognosis of patients with CRC has recently been the subject of various research [13]. Right colon cancer (RCC) has a much worse prognosis than left colon cancer (LCC). It is generally known that RCC and LCC are two distinct diseases from a clinical standpoint. In fact, RCC is more frequently diagnosed in older women with peritoneal metastases and with an undifferentiated grade than LCC, which, on the other hand, frequently exhibits liver and lung metastases [14]. The gene expression pattern is also different: RCC more frequently exhibited mucinous histologic characteristics, microsatellite instability (MSI), mutations of the RAS, BRAF, and PIK3CA genes, and had a higher TNM stage, a larger tumor size, and worse outcomes than LCC [15]. In contrast, mutations in the APC, KRAS, SMAD4, and TP53 genes, amplification of epidermal growth factor receptor (EGFR) and human epidermal growth factor receptor 2 (HER2), and overexpression of the EGFR ligands epiregulin (EREG) and antifiregulin (AREG) were more prevalent in LCC [16]. Additionally, less is known regarding the results of patients who have hepatectomy with the goal of curing SM and MM CRCLM. However, due to inconsistent findings from previous studies, the usefulness of PTL in predicting the prognosis of patients with CRCLM is still debatable.

The analysis of patients with unresectable CRCLM enrolled in six randomized studies (FIRE-3, CRYSTAL, PEAK, PRIME, CALGB 80405, and 20050181) revealed that patients with RCC had lower overall survival (OS) and progression-free survival (PFS) [17]. While some research found that LCC was linked to worse OS and recurrence-free survival (RFS) for patients with resectable CRCLM, other studies discovered that while OS was worse in RCC, RFS was similar in both RCC and LCC [18]. The major goal of the current study was to assess how PTL may affect prognosis in patients with synchronous and metachronous colorectal liver metastases.

## Materials and methods

**Figure 1:**
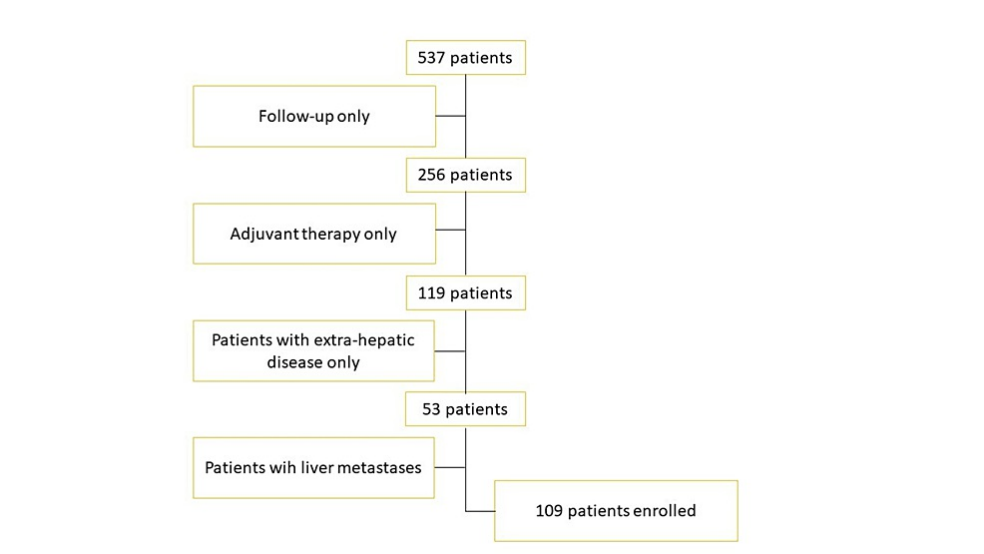
Flowchart showing the criteria for inclusion and exclusion

For this analysis, we reviewed all cases of colorectal cancer patients who had first accessed our Institute from 2016 to 2021. Figure 1 shows a flowchart of inclusion and exclusion. A total of 109 patients with CRCLM out of 537 were retrospectively identified and gathered in an electronic retrospective database pertaining to patient and disease characteristics, treatment, and outcomes. These patients received neoadjuvant chemotherapy, surgical resection followed by adjuvant chemotherapy, or chemotherapy with palliative intent. From medical records, the authors extracted every piece of data. The location, stage, and existence or absence of liver metastases at the time of the primary cancer diagnosis, as well as the date of detection of PTL and liver metastases. The enrolled patients fulfilled the subsequent inclusion requirements: Colorectal adenocarcinoma with histological confirmation; stage IV colorectal cancer with liver metastases at diagnosis or during follow-up; synchronous or metachronous liver metastases; prior adjuvant or neoadjuvant therapies in colorectal cancer stages II-IV; and prior lines of therapy using monoclonal antibodies (mAbs) and cytotoxic agents were all permitted. Patients having more than one primary tumor and those whose primary tumors were located on both the left and right sides of the body were disqualified from the analysis.

RCC was the classification given to primary cancers coming from the appendix, cecum, ascending colon, hepatic flexure, and transverse colon in the whole study population. LCCs were defined as primary tumors coming from the splenic flexure, descending colon, sigmoid colon, and rectum. Details about the timing (synchronous/metachronous), quantity, and largest tumor size of CRCLM were also collected. We regarded liver metastases found prior to surgery, during surgery, or within six months of surgery as synchronous (SM). On the other hand, liver metastases identified six months following the original tumor surgery were classified as metachronous (MM). According to PTL, patients were divided between two groups (RCC and LCC). Then, patients with metachronous hepatic metastases and patients with synchronous liver metastases originating from the left or right primary tumor were divided into two distinct groups (SM-RCC and SM-LCC) (MM-RCC and MM-LCC).

To investigate the prognostic significance of PTL in patients with hepatic SM and MM, long-term survival information and clinicopathological data were gathered and analyzed. From the time when liver metastases were diagnosed until the time that a person died from cancer or another cause, the overall survival (OS) was determined. From the time when liver metastases were diagnosed until the time that the disease relapsed, the progression-free survival (PFS) was assessed. In addition, we specifically calculated OS from the date of liver surgery to the date of death or last follow-up, and PFS from the date of liver surgery to the date of disease progression, according to the site of the primary tumor, in the subgroup of patients undergoing surgical resection of liver metastases.

The Ethics Committee accepted our study (Comitato Etico Lazio 1 issued approval 530/CE Lazio 1), and it accorded with the Declaration of Helsinki and good clinical practice. Due to the nature of the retrospective investigation, informed consent was waived, and patient data confidentiality was maintained. The study's pertinent data were assembled using descriptive statistics. Kaplan-Meier was used to create the OS and PFS curves. According to important characteristics, a Mantel-Cox log-rank test was employed to compare the survival results of different patient subgroups. To calculate the hazard ratio (HR) with a 95% CI of confirmed significant variables, a multivariate Cox regression model was used. Possible relationships were assessed using chi-square and Fisher Exact testing. Every test that was run was two-sided, which means that it was defined at a p-value less than 0.05. All of the statistical analyses were performed using the SPSS (version 21.0) statistical program (IBM Corp., Armonk, USA).

## Results

Among the 109 patients examined, 66 (60.5%) were men and 43 (39.4%) were women. The primary tumor was detected on the right side in 46 patients (42.2%) and on the left side in 63 patients (57.7%). Patients who presented SM and SM-LCC at the diagnosis were prevalent (69.7% and 42.2%, respectively). The median age was 70 yrs (range 35-91) for all patients, 72,5 yrs (range 45-84) for SM-RCC patients, 68,5 yrs (range 56-81) for MM-RCC patients, 69,5 yrs (range 35-91) for SM-RCC patients and 64 yrs (range 42-77) for MM-LCC patients. The median duration of follow-up was 21,3 months (95%CI=15,4-25,2). ≥5 hepatic metastases prevailed in the SM group (N=61; 83.5%). Table 1 provides a summary of the baseline clinicopathological features of all eligible patients.

**Table 1:** Clinicopathological characteristics of patients Abbreviations: CRCLM, colorectal cancer liver metastases; SM, synchronous metastases; MM, metachronous metastases; RCC, right-sided colon cancer; LCC, left-sided colon cancer; PTL, primary tumor location; R0, no residual disease; MSI, microsatellite instability; MSS, microsatellite stability; Monoclonal Abs, monocolonal antibodies. Fisher’s exact test was used for all categorical variables.

		CRCLM N=109	
	SM	MM	
	N(%)	N (%)	P-value
Total	76 (69.7)	33 (30.2)	-
Age (yrs)			0.14
<70	33 (30.2)	20 (18.3)	
≥70	43 (39.4)	13 (11.9)	
Gender			0.09
Female	34 (31.1)	9 (8.2)	
Male	42 (38.5)	24 (22.0)	
RAS status			0.32
Wild type	31 (28.4)	10 (9.1)	
Mutant Type	24 (22.0)	14 (12.8)	
BRAF status			1.00
Wild type	14 (12.8)	8 (7.3)	
Mutant type	1 (0.9)	0 (-)	
PTL			0.40
RCC	30 (27.5)	16 (14.6)	
LCC	46 (42.2)	17 (15.5)	
MSI status			0.11
MSI	0 (0)	2 (1.8)	
MSS	18 (16.5)	8 (7.3)	
No. liver metastases			0.00
≥5	60 (55.0)	12 (11.0)	
<5	16 (14.6)	21 (19.2)	
Metastatic disease sites			0.67
Liver only	37 (33.9)	18 (16.5)	
Liver + other	39 (35.7)	15 (13.7)	
Maximum liver metastases size (cm)			0.25
≥5	25 (22.9)	7 (6.4)	
<5	51 (46.7)	26 (23.8)	
Liver-resected patients			0.11
Yes	12 (11.0)	11 (10.0)	
No	64 (58.7)	22 (20.1)	
R0 resection			1.00
Yes	9 (39.1)	9 (39.1)	
No	3 (13.0)	3 (13.0)	
Patients who relapse after liver resection			0.68
Yes	8 (34.7)	6 (26.0)	
No	4 (17.3)	5 (21.7)	
Chemotherapy regimen			0.03
Fluoropirimidine alone	12 (15.7)	4 (12.1)	
Doublet chemotherapy	49 (64.4)	23 (69.6)	
Triplet chemotherapy	3 (3.9)	1 (3.0)	
Monoclonal Abs			0.26
Cetuximab use	5 (6.5)	1 (3.0)	
Panitumumab use	19 (25.0)	5 (15.1)	
Bevacizumab use	22 (28.9%)	10 (30.3)	
Dead or alive			0.46
Dead	8 (7.3)	2 (1.8)	
Alive	27 (24.7)	17 (15.5)	

**Figure 2:**
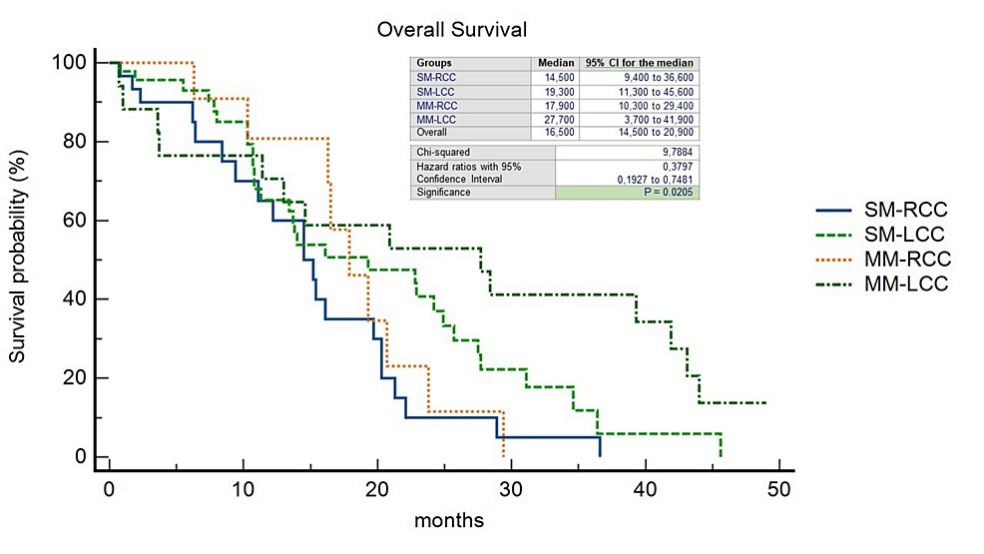
Kaplan-Meier curves of Overall Survival stratified by primary tumor location and timing of liver metastases. SM-RCC, synchronous metastases from right-sided colon cancer; SM-LCC, synchronous metastases from left-sided colon cancer; MM-RCC, metachronous metastases from right-sided colon cancer; MM-LCC, metachronous metastases from left-sided colon cancer

**Figure 3:**
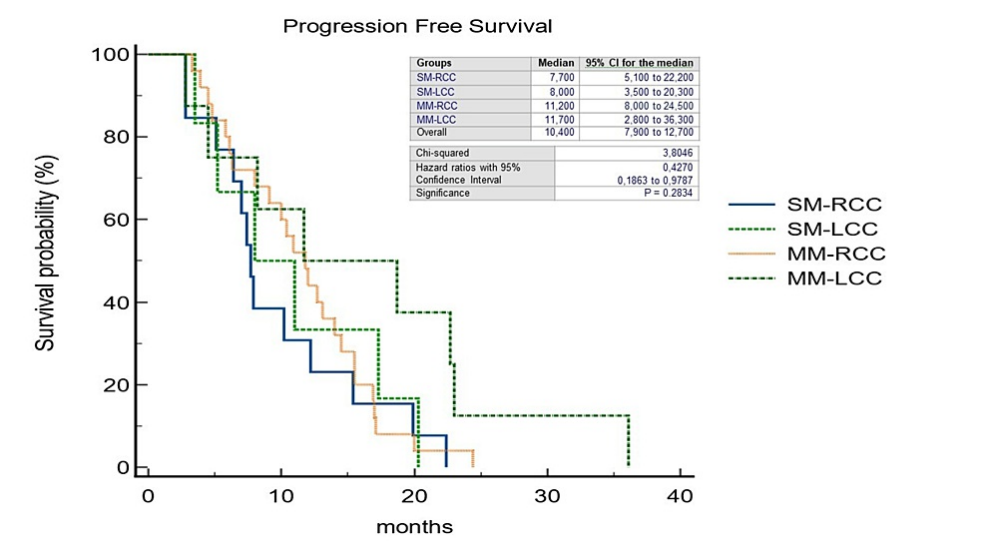
Kaplan-Meier curves of Progression-Free Survival stratified by primary tumor location and timing of liver metastases. SM-RCC, synchronous metastases from right-sided colon cancer; SM-LCC, synchronous metastases from left-sided colon cancer; MM-RCC, metachronous metastases from right-sided colon cancer; MM-LCC, metachronous metastases from left-sided colon cancer

**Figure 4:**
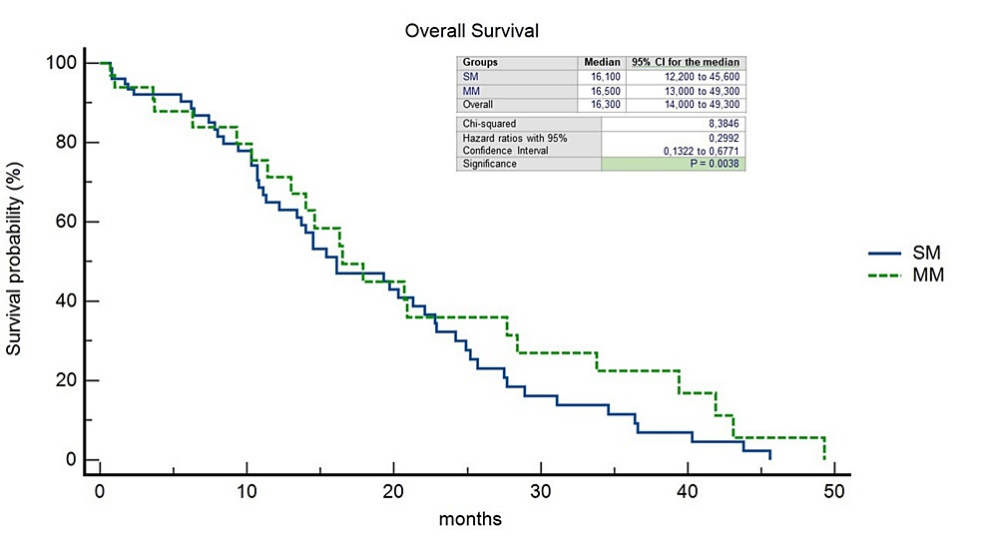
Kaplan-Meier curves of Overall Survival stratified by timing of liver metastases. SM, synchronous metastases; MM, metachronous metastases

**Figure 5:**
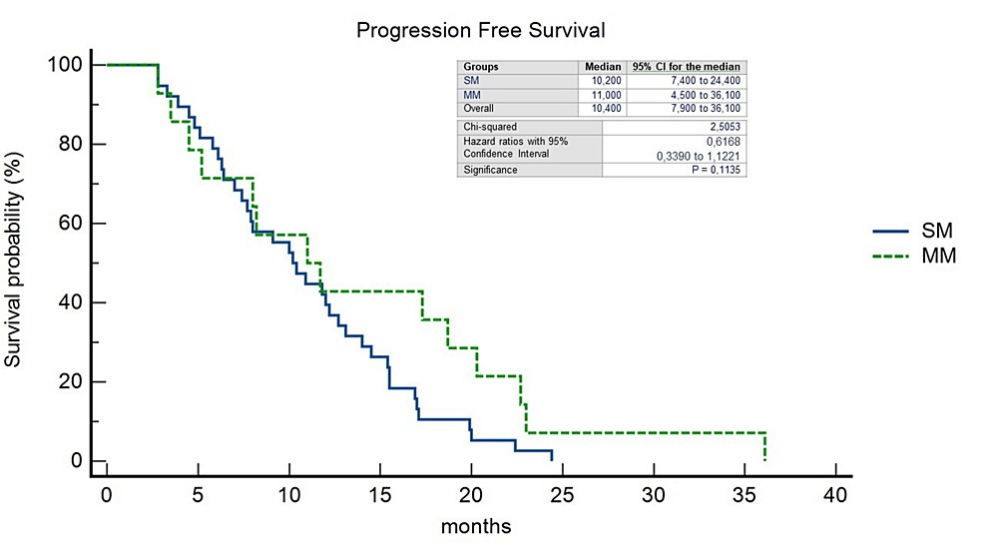
Kaplan-Meier curves of Progression-Free Survival stratified by timing of liver metastases. SM, synchronous metastases; MM, metachronous metastases

The pattern of recurrence differed between patients with LCC and RCC: 32 out of 63 patients with LCC (50,7%) experienced a recurrence, compared to 18 out of 46 patients with RCC (39.1%) (p=0.49). In the RCC group, the liver-limited, both extrahepatic and intrahepatic, and the widespread recurrence rate was 38.8%, 50%, and 11.1%, respectively; on the other hand, in the LCC group, it was 40.6%, 43.7%, and 15.6%, respectively. 21% of all patients underwent CRCLM resection (R0=78.2%); within this group, 18.1% was SM-RCC (R0=25%), 13.6% MM-RCC (R0=6.2%), 36.3% SM-LCC (R0=37.5%) and 31.8% MM-LCC (R0=37.5%). In a non-statistically significant manner, we observed a double rate of patients unresponsive to standard systemic antineoplastic treatments in the SM group (35.8% vs. 17.9% of the MM group) (p=0.27) and at the same time a disease control rate in favor of SM patients (55.2% vs. 19.4% of the MM patients) (p=0.76). We observed a significantly longer median OS in patients with MM-LCC compared with the other groups (27.7 months; HR=0.3797; 95%CI=0.19-0.74; p=0.0205) (Figure 2) as well as median PFS although the latter was not statistically significant (11.7 months; HR=0.42; 95%CI=0.18-0.97; p=0.28) (Figure 3). The median OS, regardless of PTL, was found to be higher in the MM group (16,5 months vs. 16,1 months; HR=0,29; 95%CI=0,13-0,67; p=0.0038) as well as PFS (11 months vs. 10,2 months; HR=0,61; 95%CI=0,33-1,12; p=0.11) (Figures 4, 5).

**Figure 6:**
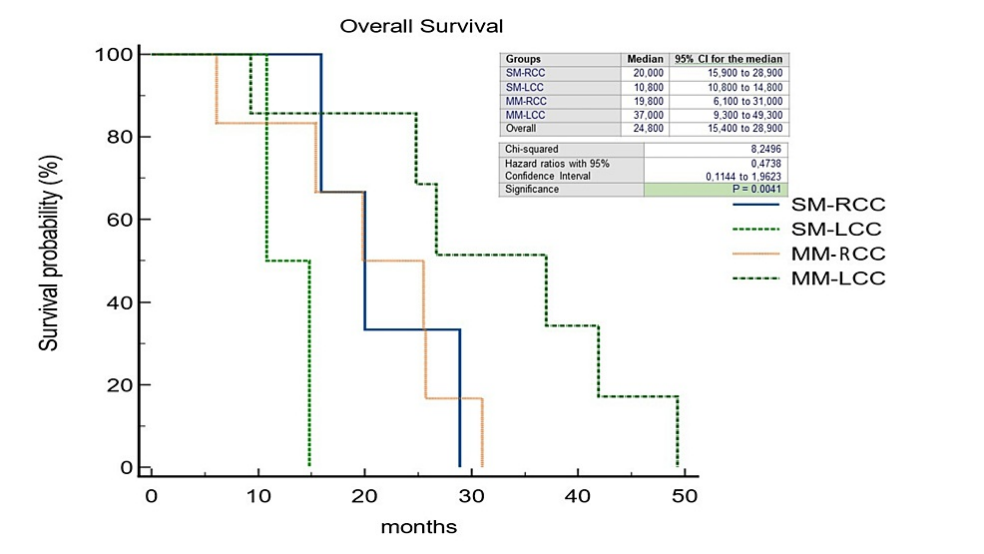
Kaplan-Meier curves of Overall Survival in patients after curative-intent surgery for colorectal cancer liver metastases stratified by primary tumor location and timing of liver metastases. SM-RCC, synchronous metastases from right-sided colon cancer; SM-LCC, synchronous metastases from left-sided colon cancer; MM-RCC, metachronous metastases from right-sided colon cancer; MM-LCC, metachronous metastases from left-sided colon cancer

**Figure 7:**
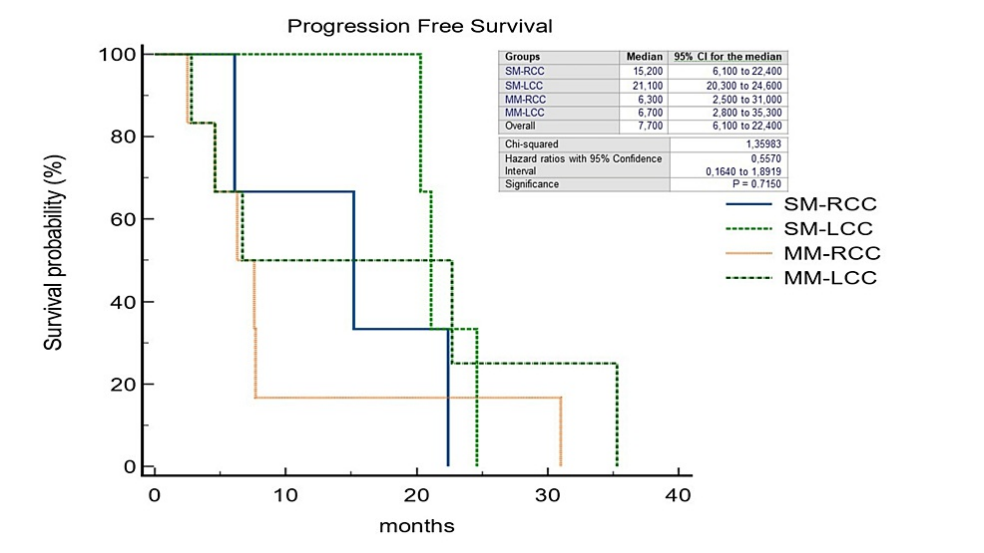
Kaplan-Meier curves of Progression-Free Survival in patients after curative-intent surgery for colorectal cancer liver metastases stratified by primary tumor location and timing of liver metastases. SM-RCC, synchronous metastases from right-sided colon cancer; SM-LCC, synchronous metastases from left-sided colon cancer; MM-RCC, metachronous metastases from right-sided colon cancer; MM-LCC, metachronous metastases from left-sided colon cancer

Also in patients undergoing liver surgery, a noteworthy median OS was shown to be significantly in favor of patients with metachronous liver metastases from the primary left tumor (37.0 months; HR=0.47; 95%CI=0.11-1.96; p=0.0041) (Figure 6). Finally, although consisting of a small number, in a not significant manner, we observed that in patients who underwent surgery because of the occurrence of liver metastases synchronous to left-located colorectal cancer, the median PFS was longer than in the other study groups (21.1 months; HR=0.55; 95%CI=0.16-1.89; p=0.71) (Figure 7).

## Discussion

A strong body of evidence suggests that the sidedness of colorectal cancer is a distinct predictive factor for survival. Patients with the right primary tumor site tend to present, at the onset, with a more extensive stage of disease, and with worse responses to treatments. Due to studies' inconsistent findings, the relevance of PTL in predicting a patient's prognosis for colorectal cancer with liver metastases is still debatable in this context. Our real-world analysis's objective was to assess the prognostic significance of the initial tumor site in patients with metastatic colorectal cancer and, more specifically, in patients with synchronous and metachronous liver secondaries. In our study, with the limitation of the retrospective data, we noticed a potential prognostic relationship between the primary colon cancer location and the timing of liver metastases. Both parameters are very important for patient’s prognosis. In detail, we showed that patients with MM-LCC have the best clinical outcomes. Patients with SM-RCC had the worst results, followed by SM-LCC and MM-RCC. We noticed the same result, although in a smaller number of patients, even in the subgroups who underwent hepatic resection.

The prognostic value of distinction of liver metastases detected synchronously and metachronously has been highlighted by several studies [19, 20]. Among others, we mention Adam et al. who pointed out that MM should be considered as having both better survival and biology [21]. The authors of Colloca et al.'s study looked at patient outcomes based on the time of metastases as well as the various tumor characteristics connected to SM and MM and came to the conclusion that patients with SM have a bad prognosis (18.5 vs. 62.5 months) [22]. Not yet fully defined are the parameters used to differentiate temporally between SM and MM: in general, however, there has been a tendency to observe in the studies conducted so far, a prolonged OS in patients with MM although evidence that the synchronous-metachronous split has a definitive value in terms of prognosis is still lacking. In this regard, Furukawa et al. investigated the potential prognostic significance of liver SM and MM not undergoing surgical resection, finding no difference in OS. Specifically, the outcomes of the multivariate analysis revealed that the Glasgow Prognostic Score (GPS) may be an effective prognostic indicator for patients who had unresectable CRCLM, including SM and MM [23].

Some studies have defined liver metastases as synchronous when they occur within three months of the diagnosis of the primary tumor; others when metastases were present up to a year after diagnosis; others still have not further deepened the distinction adopted. In our investigation, metastases were categorized as synchronous up to six months after a CRC diagnosis and metachronous if discovered after that time. The rate of synchronous liver metastases reported in our study was 69.7%, which is much higher than the incidence of SM reported in other studies, which ranged from 14.5% to 24.5%. [24]. These discrepancies in the incidence of SM could be the result of differences in defining liver metastases in different countries. Or the incidence can be higher thanks to the evolution of instrumental imaging with the realization of a more detailed diagnosis in the early stages of the diagnostic process. As a result, the increase in the incidence of SM would favor the approach to the most aggressive and targeted treatments possible.

Due to the prognostic and predictive distinctions between RCC and LCC, it is now well recognized and understood that CRC is no longer regarded as a single pathology. Among clinical outcomes, survival in RCC is significantly worse than in LCC. As the pathways implicated in the neoplastic development of CRC are based on anatomical location, RCC and LCC are two embryologically separate entities. A poor response to chemotherapy may be caused by the greater prevalence of BRAF and KRAS mutations and high levels of microsatellite instability found in RCC. The liver is the primary location for metastases in 30-50% of people with CRC because of its draining from the gastrointestinal tract. At the time of diagnosis, 50% of CRC patients have liver SM. Due to the progression of the disease, only 10 to 30 percent of individuals that meet the criteria for resection at initial presentation are typically qualified for liver resection. The only potentially curative treatment for CRCLM is still liver resection, which has 10-year OS rates as high as 16% and a five-year survival rate that is currently above 50% in most series. Unfortunately, within two years, the condition will return in roughly 75% of individuals. The prognosis is poor if untreated, with a five-year survival rate that is almost zero according to historical statistics [25].

More and more patients can gain from down-staging techniques and, in the end, from a curative resection, which is frequently followed by positive long-term results. The current response rates for down-staging are over 50% when systemic chemotherapy and immunotherapy are combined. On radically deleted CRCLMs, contentious statistics were provided. Patients with CRCLM from RCC have been linked to worse OS in numerous studies, while there are conflicting findings about PFS [26, 27]. Following CRCLM surgery, the location of the original tumor on the right side is linked to a worse OS but does not seem to alter PFS, which is consistent with our data. The RCC liver metastases appear to recur less frequently but in a more aggressive manner. To support this, our real-world analysis found a different pattern of recurrence between LCC and RCC patients: 39.1% of RCC group reported a recurrence compared to 50.7% of the reverse group. Similar findings are available from Garajova et al. and Russolilo et al. [28, 29]. However, some authors argue different responsiveness and sensitivity of patients with SM, probably as chemo-naïve, with higher response rates than the other groups of patients [30].

In light of the above-mentioned, we propose both the timing of occurrence of liver metastases and PTL as important parameters for patients’ prognosis. We also suggest that they could be helpful as stratifying factors in CRCLM peri-operative multidisciplinary management and in the systemic therapies decision-making process in advanced disease. There are, however, different limitations in our study. Due to the retrospective design and small sample size from a single institution, care must be used when interpreting and extrapolating the findings. To clarify the impact on prognosis, we suggest the need for a meta-analysis or a systematic review to finally evaluate the prognostic correlation between PTL and synchronous vs. metachronous liver metastases from colon cancer.

## Conclusions

Our retrospective real-world study's findings imply that whether a patient with liver SM or MM undergoes hepatic surgical resection or not may be affected by the location of their initial colorectal cancer in terms of prognosis. The liver is confirmed to have the largest prevalence of SM lesions and compared to MM, they have a poorer prognosis. Even though it involved a small sample of patients, our study was able to confirm that in patients with CRCLM, the right side of the primary tumor and the synchronous onset time of liver metastases may be prognostically correlated, in terms of outcomes, in a worse survival in both the disease not amenable to hepatic resection and in the operated patients. Additional research is required in this regard.
